# Raman fingerprints as promising markers of cellular senescence and aging

**DOI:** 10.1007/s11357-019-00053-7

**Published:** 2019-02-04

**Authors:** Lisa Liendl, Johannes Grillari, Markus Schosserer

**Affiliations:** 1 Department of Biotechnology, University of Natural Resources and Life Sciences, Vienna, 1190 Vienna, Austria; 2 Evercyte GmbH, 1190 Vienna, Austria; 3 Christian Doppler Laboratory on Biotechnology of Skin Aging, 1190 Vienna, Austria

**Keywords:** Raman microspectroscopy, Cellular senescence, Senolytic compounds, Skin aging, Label-free imaging, Biomarker

## Abstract

Due to our aging population, understanding of the underlying molecular mechanisms constantly gains more and more importance. Senescent cells, defined by being irreversibly growth arrested and associated with a specific gene expression and secretory pattern, accumulate with age and thus contribute to several age-related diseases. However, their specific detection, especially in vivo, is still a major challenge. Raman microspectroscopy is able to record biochemical fingerprints of cells and tissues, allowing a distinction between different cellular states, or between healthy and cancer tissue. Similarly, Raman microspectroscopy was already successfully used to distinguish senescent from non-senescent cells, as well as to investigate other molecular changes that occur at cell and tissue level during aging. This review is intended to give an overview about various applications of Raman microspectroscopy to study aging, especially in the context of detecting senescent cells.

## Introduction

Demographic changes in the industrialized world lead to an increased occurrence of medical conditions for which biological age is the major risk factor (GBD 2015 DALYs and HALE Collaborators 2016; GBD 2015 Mortality and Causes of Death Collaborators 2016). In addition to arising complications and onset of multimorbidity due to decreased resilience, frailty severely limits the quality of life at advanced age. Thus, a better understanding of biological aging processes and identification of markers will promote the design of interventions, which target biological aging and thereby increase fitness, decrease frailty, and improve resilience at advanced age (Bellantuono 2018; Cardoso et al. 2018; Figueira et al. 2016).

One of these processes, cellular senescence, is defined not only as an irreversible growth arrest induced by either serial passaging, which causes the shortening of telomeres to a critical length (replicative senescence) (Bodnar et al. 1998; Hayflick and Moorhead 1961), or by exposure to stress (stress induced premature senescence = SIPS) (Toussaint et al. 2002), but also as a consequence of chemo- and radiation therapy (Demaria et al. 2017; Schosserer et al. 2017) or oncogene activation (Collado and Serrano 2006). Senescent cells accumulate in the body during normal aging and occur predominantly at sites of age-associated pathologies, which include atherosclerosis (Erusalimsky and Kurz 2005; Gorenne et al. 2006; Minamino 2002; Vasile et al. 2001), osteoporosis (Kassem and Marie 2011), neuroinflammation (Bitto et al. 2010), and liver cirrhosis (Wiemann et al. 2002). While considered being a beneficial tumor suppressor mechanism in the young (Campisi et al. 2011; Campisi 2005), cellular senescence is by now well accepted to contribute to in vivo aging (Baar et al. 2017; Baker et al. 2016, 2011; Xu et al. 2018) and even tumor progression in the elderly (Campisi et al. 2011; Campisi 2005). These deleterious effects are caused at least in part by the senescence-associated secretory phenotype (SASP) (Coppe et al. 2010), which was already shown to promote chronic inflammation and thereby fuel several aging-associated pathologies including atherosclerosis, kidney fibrosis, and cancer progression (Demaria et al. 2017; Schosserer et al. 2017). Thus, one of the major goals of current aging research is the development of compounds that specifically eliminate senescent cells (“senolytics”) or inhibit the SASP and thereby alleviate deleterious effects caused by senescent cells (Baar et al. 2017; Xu et al. 2018; Zhu et al. 2017, 2015).

However, although a prerequisite for screening and evaluation of senolytic compounds, the detection of senescent cells, especially in vivo, is still one of the challenges in the field. Currently, flattened cell morphology, activation of p16^INK4a^ (Baker et al. 2016; Tchkonia et al. 2013) and p53 (Tchkonia et al. 2013), activity of SA-β-Galactosidase (Debacq-Chainiaux et al. 2009), staining with Sudan Black B (Georgakopoulou et al. 2013), presence of ɣH2AX foci at the telomeres (Fumagalli et al. 2014) and senescence-associated heterochromatin foci (Narita et al. 2003), High Mobility Group Box 1 (HMGB1) secretion (Davalos et al. 2013), and growth arrest as measured by BrdU-incorporation (Lämmermann et al. 2018) are considered to be senescence markers. The drawback is that none of them is specific for senescence and some of them can only be detected in vitro. Therefore, combinations of these markers have to be used. Raman microspectroscopy could thus offer a non-invasive and label-free method that allows to monitor the progression of senescence in real time in vitro and in vivo.

## Raman microspectroscopy distinguishes cellular states in a label-free and non-invasive manner

Raman spectroscopy is based on the interaction between light that is focused on a sample and the chemical bonds within the material to be analyzed. Compared to elastic or Rayleigh scattering, inelastic or Raman scattering is a rare and comparatively weak phenomenon. Depending on the direction of the energy shift (Raman shift in cm^−1^), scattered electrons are either at lower (Stokes Raman) or higher (anti-Stokes Raman) energy levels (Raman 1928).

**Fig. 1:**
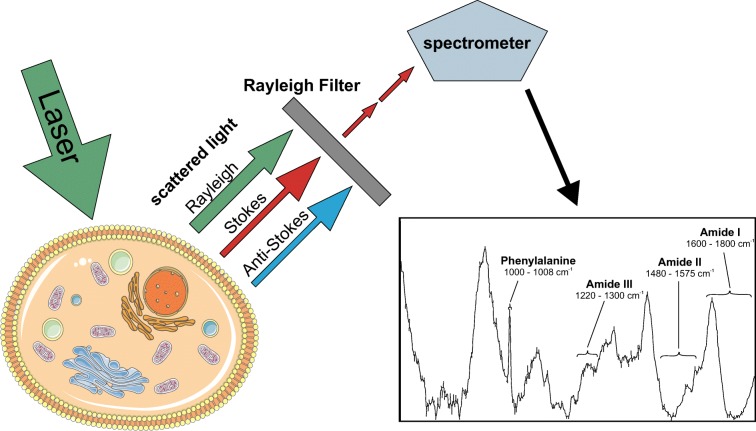
Raman microspectroscopy of cells. Photons emitted from a laser light source are differentially scattered by chemical bonds of cellular constituents. Rayleigh and anti-Stokes scattered light is filtered, and remaining Raman Stokes scattered photons are recorded by the spectrometer. A typical Raman spectrum of mammalian cells is shown. Spectral regions explaining the most prominent differences between cellular states are depicted

Modern Raman microspectrometers consist of a confocal microscope equipped with one or more lasers, an efficient longpass filter to remove highly abundant Rayleigh-scattered light, a spectrometer with different gratings, and a sensitive CCD line detector (Fig. 1). UV and blue lasers go along with high energy, which might damage biological samples, and induce significant levels of autofluorescence. Thus, green and red lasers (e.g., 532 nm or 785 nm) are most commonly used for the analysis of cells and tissues. Most current Raman microscpectrometers offer automated mapping applications, whereby the laser scans over the specimen and a spectrum is recorded at every single pixel to generate a multi-dimensional hyperspectral image. While acquiring spectra is relatively simple, data processing poses a major challenge and usually consists of background removal and normalization steps, followed by multivariate statistic approaches including principal component analysis (PCA), linear discriminant analysis (LDA), classical least square (CLS) fitting, multivariate curve resolution (MCR), among others (Butler et al. 2016; Notingher et al. 2005).

Raman spectra from biological materials, typically recorded in the region of 400–2000 cm^−1^ (Fig. 1), provide chemical fingerprints detecting even subtle changes in the biochemical composition of cells (Beattie et al. 2013; Brauchle and Schenke-Layland 2013; Charwat et al. 2015; Rösch et al. 2006; Swain and Stevens 2007), tissues (Ashtikar et al. 2013; Bocklitz et al. 2013; Movasaghi et al. 2007), and whole organisms (Lau et al. 2012). The advantage of Raman microspectroscopy compared to traditional staining approaches lies in the fact that this technique can be used on living specimen without prior fixation and does not require any label that might interefere with normal physiology. Raman signatures of in vitro cultured cells were already successfully recorded and used for the characterization and identification of various specific cell types, as for example for endothelial (Szafraniec et al. 2018) and human lung (Surmacki et al. 2018) cell lines. Our lab was also able to distinguish different Chinese Hamster Ovary (CHO) host and production cell lines by Raman microspectroscopy (Prats Mateu et al. 2017). In chondrocytes (Pudlas et al. 2013), hematopoetic stem cells (Ilin et al. 2015), and hematopoetic progenitor cells (Choi et al. 2018), it has been shown that Raman microspectroscopy is capable of monitoring the dynamic process of cell differentiation. Also, different cellular states, such as apoptosis and necrosis, were successfully distinguished (Brauchle et al. 2014b), and cell progression through mitosis was followed by Raman microspectroscopy (Matthäus et al. 2006).

Biochemical deviations occuring in cancer have been extensively studied using Raman spectroscopy not only at cellular level in vitro (Brauchle et al. 2014a; Duraipandian et al. 2018; Lee et al. 2018; Managò et al. 2018; Terentis et al. 2013), but also in tissues ex vivo (Bocklitz et al. 2013; Santos et al. 2016). These promising results pave the way for clinical use of Raman spectroscopy for analysis of extracted specimen and identification of markers for tumor resections (Santos et al. 2017; Shipp et al. 2018). A fiber-optic Raman probe was already used during brain surgery and allowed differentiation between cancer and healthy tissue (Jermyn et al. 2015). Another putative clinical application of Raman spectroscopy is the detection of fragility fractures by using Spatial Offset Raman Spectroscopy (SORS) (Buckley et al. 2015).

## Raman microspectroscopy enables distinction of senescent and non-senescencent cells

Only few studies were conducted to investigate cellular senescence using Raman microspectroscopy so far. Bai and coworkers acquired Raman signatures of mesenchymal stem cells obtained from human umbilical cord tissue during serial passaging (Bai et al. 2015). The authors found that the ratio of peaks at 1157 cm^−1^ vs. 1174 cm^−1^, both corresponding to vibrations of proteins, could serve as a marker for late population doubling levels (PDLs). Other notable, but not significant, differences between Raman spectra of late and early PDL cells were found within the amide II (1480–1575 cm^−1^) region.

Eberhardt and coworkers analyzed four different human dermal fibroblast cell strains using Raman spectroscopy as well as Fourier transform infrared spectroscopy (FTIR) (Eberhardt et al. 2017a). Comparing Raman signatures of early PDLs, middle PDLs, and senescent cells, peak intensities at 1580 cm^−1^ and 1658 cm^−1^ assigned to nucleic acids and proteins, respectively, were found to be decreased, while lipid associated peaks at 1732 cm^−1^, 2850 cm^−1^, and 2930 cm^−1^ were increased in senescent cells. Partial least squares-linear discriminant analysis (PLS-LDA) was able to distinguish these three groups. Analysis of the difference spectra obtained through PLS-LDA again revealed changes in the amide I region (1600–1800 cm^−1^), at high wavenumbers (> 2800 cm^−1^), as well as in the amide III region (1220–1300 cm^−1^), and below 1200 cm^−1^. Raman-based classification models set up for each cell strain separately revealed an overall sensitivity of 93% and specificity of 90%, although outcomes from the four cell strains differed. Senescence was confirmed by morphological changes, cell proliferation in different PDLs, as well as SA-β-galactosidase activity.

In another study, Eberhardt and coworkers expanded their Raman and FTIR-based detection of senescent dermal fibroblasts towards a 3D model of human skin (Eberhardt et al. 2017b). Fibroblast-derived matrices (FDM) were built by seeding fibroblasts in PDL 4 and PDL 20 for the young and senescent model, respectively. In 3D, Raman peaks between 600 and 900 cm^−1^ and a peak at 1260 cm^−1^ associated with the amide III region were decreased in senescent cells. The spectral region between 930 and 1230 cm^−1^ showed increased intensities in the spectra of senescent cells. Comparison of fibroblasts from passages 4, 7, and 20 in 2D culture showed alterations below 1250 cm^−1^ and in the amide I and II region. PLS-LDA for cells cultivated in 2D and 3D revealed differences in the amide I and II region, as well as at 788 cm^−1^, a peak that can be assigned to ring breathing modes in nucleic acids. A classification model trained with proliferating and senescent cells grown in 3D was then indeed able to predict these cellular states in 2D culture. However, vice versa, classification of 3D data was not successful when a 2D training set was used, underlining the fact that other differences became more obvious in the 3D environment. Senescence was confirmed by SA-β-galactosidase staining.

Oncogene-induced senescence was studied in MCF-7/NeuT cells (Mariani et al. 2010). Senescence was induced by doxycycline treatment, leading to oncogenic ErbB2 overexpression and consequently p21 induction (Trost et al. 2005). Raman spectra of nuclei from senescent cells showed a single peak at 1652 cm^−1^, whereas two peaks at 1652 cm^−1^ and 1666 cm^−1^ were found in control cells. These two peaks were assigned to *cis* and *trans* unsaturated fatty acid isomers, respectively. However, as the amide II band is also located in this region, interpretation of the signal beeing protein-derived seems also possible. Mariani and coworkers concluded that mainly *cis* isomers can be found in senescent cells, leading to instabilities in the nuclear membrane. Furthermore, peaks at 1313 cm^−1^ and 1339 cm^−1^ assigned to glycoproteins were found in control, but not in senescent cell spectra. Accordingly, mRNA levels of nuclear pore complex glycoprotein Nucleoporin 210 (NUP210) were significantly decreased in senescent cells. The assignment of the glycoprotein peak was based on a publication measuring Raman spectra from an isolated antifreeze glycoprotein (Tomimatsu et al. 1976). In case of cell-based Raman signatures, proteins and nucleic acids might also be worth considering for being the source of chemical interactions located in that wavenumber region.

As summarized in Table 1, in all the studies comparing Raman signatures between young and senescent cells (Bai et al. 2015; Eberhardt et al. 2017a, b; Mariani et al. 2010), peaks assigned to the amide II region were subject to substantial changes. The amide II band between 1480 and 1575 cm^−1^ refers to C–N stretching and N–H bending occuring in peptides (Movasaghi et al. 2007). The amide I band between 1600 and 1800 cm^−1^, related to C=O stretching and the amide III band from 1220 to 1300 cm^−1^ depicting C–N stretching and N–H bonding (Movasaghi et al. 2007) also constantly recured with the exception of the studies done by Bai and coworkers. Differences in the biochemical fingerprint between young and senescent cells could thus be explained by varying occurences of proteins. However, chemical interactions associated with glycoproteins, lipids, and nucleic acids also contribute to the variations located in the range of the three dominant amide bands.

**Table 1:** Most prominent peaks and spectral regions contributing to differences in spectra from senescent versus non-senescent cells. The corresponding studies, as well as peak assignments differing from Movasaghi et al. (2007), are indicated

Spectral region	Peak assignment (Movasaghi et al. 2007)	Bai et al. (2015)	Eberhardt et al. (2017a)	Eberhardt et al. (2017b)	Mariani et al. (2010)
600–900 cm^−1^	Ring breathing modes in nucleic acids (among others)			x	
788 cm^−1^	Ring breathing modes (nucleic acids)			x	
930–1230 cm^−1^	Proteins, lipids, glycogen, glucose, nucleic acids			x	
1156/7 cm^−1^	C–C, C–N stretching (proteins)	x			
1174 cm^−1^	C–H bending (tyrosine, phenylalanine)	x			
1220–1300 cm^−1^	Amide III region: C–N stretching, N–H bonding		x	1260 cm^−1^	
1313 cm^−1^	CH_3_CH_2_ twisting (lipids)				x (glycoproteins)
1339 cm^−1^	CH_3_CH_2_ twisting (lipids), C–C stretching (phenyl)				x (glycoproteins)
1480–1580 cm^−1^	Amide II region: C–N stretching, N–H bending, ring breathing modes (nucleic acids)	x	1580 cm^−1^ (nucleic acids)	x	
1652 cm^−1^	C=C stretching (lipids)				x (*cis* unsaturated fatty acids)
1666 cm^−1^	C=C stretching (proteins)				x (*trans* unsaturated fatty acids)
1600–1800 cm^−1^	Amide I: C=O stretching, C=C stretching (proteins, lipids)		1658 cm^−1^ (proteins), 1732 cm^−1^ (lipids)	x	
2850 cm^−1^	CH_2_ stretching (lipids, fatty acids)		x		
2930 cm^−1^	CH_2_ stretching (lipids)		x		

## Raman spectroscopy is able to visualize molecular changes occurring in skin aging

Raman-based in vivo investigations have been performed to analyze age-related changes in human skin and its components. Especially the stratum corneum (SC), the outermost part of the epidermis, has been subject to these studies (Boireau-Adamezyk et al. 2014; Choe et al. 2018; Egawa and Tagami 2008). Differences between young and aged female subjects regarding water content in the SC of forearm skin were found using Raman signatures (Egawa and Tagami 2008). Also, changes in the barrier function of SC were observed, especially a decreased lipid/protein ratio, as well as an increased transepidermal water loss with age and an increased SC thickness, though the condition of the barrier function also strongly depended on the site of measurement (Boireau-Adamezyk et al. 2014). Contradictorily, another Raman-based study, including subjects from a smaller age range, showed that the lipid/protein ratio stayed constant with increasing age, while the expansion in SC thickness was confirmed (Choe et al. 2018). The dermis, the skin layer underneath the epidermis, was examined regarding the water content by using a prediction model, followed by Raman-based analysis, pointing out higher water content in the dermis of healthy aged and diabetic women compared to healthy young women (Téllez et al. 2015).

Special interest was given to photoaged skin which was investigated ex vivo (Gniadecka et al. 1998; González et al. 2012). Raman spectra recorded from chronologically aged skin as well as photoaged skin obtained by punch biopsies from a total of 20 individuals, showed a shift towards lower wavenumbers in the amide I band compared to young skin. In photoaged skin, the amide III region and C–H stretching bands higher than 2800 cm^−1^ were also shifted towards lower wavenumbers, possibly indicating an increase in protein folding in photoaged skin. However, in chronologically aged skin, only the peak at 1658 cm^−1^ in the amide I region was different from young individuals (Gniadecka et al. 1998). Raman spectroscopy was used to study intrinsic aging and photoaging in vivo in 15 subjects between 28 and 82 years of age, divided into three different groups (de Vasconcelos Nasser Caetano et al. 2017). The authors pointed out the proline-hydroxyproline region (intensities of peaks at 855 cm^−1^ and 938 cm^−1^) as suitable for the evaluation of intrinsic skin aging. Similarly, Villaret and coworkers showed that the 938/922 cm^−1^ peak ratio was decreased in spectra from aged photo-protected skin compared to aged exposed, young photo-protected, and young exposed skin obtained via punch biopsies from 14 female individuals (Villaret et al. 2018). Results from another study (Nguyen et al. 2013) show that the proline-hydroxyproline region, more specifically the 938/922 cm^−1^ peak ratio, turned out not to be able to distinguish between resected skin samples from the dermis of four females classified in two different age groups (40 years, 70 years). Nguyen and coworkers also found that the 1658/1668 cm^−1^ peak ratio, assigned to reflect interactions of water with collagen, was able to differentiate between the two age groups. However, considering the biological variability, the relatively small number of analyzed samples in these studies might not be sufficent to draw generalizable conclusions. Furthermore, the penetration depth of Raman probes for in vivo use is relatively low, allowing analysis of just the upper skin layers.

Interestingly, C–H stretching bands that were found to be shifted in photoaged skin (Gniadecka et al. 1998) were among the regions that also contributed to differences in the spectra of senescent cells in comparison to non-senescent cells (Eberhardt et al. 2017a). Furthermore, the amide I band was responsible for spectral differences at both cellular (Eberhardt et al. 2017a, b) and tissue level (Gniadecka et al. 1998; Nguyen et al. 2013).

## The application of Raman spectroscopy to study aging in various tissues

Apart from studies in the skin, research in the field of ophthalmology already made use of Raman microspectroscopy for studying processes that occur during aging, when analyzing dried human Bruch’s membranes for the quantification of advanced glycation end products (AGEs) and advanced lipoxidation end products (ALEs) that accumulate with age (Beattie et al. 2013; Glenn et al. 2007). Resonance Raman spectroscopy, a specialized Raman technique, was used to investigate age-related effects on macular pigment optical density (MPOD) (Obana et al. 2014) as well as differences in carotenoid levels in healthy subjects compared to patients with age-related macular degeneration (Bernstein et al. 2002).

Other studies focused on Raman-based analysis of bone tissue (Ager et al. 2005; Akkus et al. 2003; Gamsjaeger et al. 2010; Milovanovic et al. 2018; Toledano et al. 2018), providing insights into compositional changes that occur during aging. Ager and coworkers used deep-ultraviolet Raman spectroscopy and found significant age-related differences in the shape and intensity of the amide I band from excised cortical bones in humans (Ager et al. 2005). As reported recently, AGEs might also contribute to the aging process of bones and show a specific Raman signal (Toledano et al. 2018). Raman spectroscopy has also been applied to investigate age-related structural changes of human teeth (Ager et al. 2006; Tramini et al. 2001). Similarly, Tramini and coworkers found that the chronological age of an individual could be predicted by analysis of the dentin’s Raman spectra (Tramini et al. 2001). Apart from the importance of understanding aging-related mechanisms, this approach might also be of interest for forensic investigations.

The same applies to another study showing successful classification into three age groups (< 1 year, 11–13 years, 43–68 years), based on Raman spectra of human peripheral blood (Doty and Lednev 2018). Apart from blood, other biofluids might provide suitable substrates for Raman-based investigation of aging phenomena as well. Erythrocyte aging has recently been studied with the help of Raman spectroscopy, revealing changes in lipids and membrane proteins (Dinarelli et al. 2018).

Multivariate statistics were able to classify spectra from human oral buccal mucosa into young and physiologically aged individuals, without inferering with the classification of Raman spectra based on tobacco-related changes (Sahu et al. 2012). Alterations in lipid composition due to aging were also examined in murine perivascular adipose tissue using Raman microspectroscopy and a Raman fiber optic probe (Czamara et al. 2018). Aging-related oxidative damage in mouse oocytes leading to developmental abnormalities was studied via Raman microspectroscopy aiming towards the use of this method in assisted reproductive treatment in humans (Bogliolo et al. 2013).

When comparing these data to the Raman-based investigation of skin aging and cellular senescence, it becomes evident that peaks responsible for the differences in the analyzed spectra are frequently located in the three prominent amide regions, depicting mostly proteins, lipids, and nucleic acids.

## Summary and perspectives

As shown here, the field of aging research has just begun to make use of the label-free, non-invasive technology of Raman microspectroscopy. For studying Raman fingerprints of senescent cells, caution must be given to the precise definition and characterization of the senescent state, which was neglected by some of the previous studies complicating their interpretation. Furthermore, as senescence is by now considered to occur progressively and to show heterogeneity between indiviual cells and tissues (Hernandez-Segura et al. 2017), it would be interesting to compare early to late senescent cells during development of the characteristic SASP. Coupling Raman microspectroscopy to microfluidic systems, as reviewed by Li and coworkers (Li et al. 2012), will pave the way for investigation of heterogeneity within a large cell population. It also remains to be seen if Raman bands explaining the differences between senescent and non-senescent cells vary between different cell types and tissues, and if these fingerprints might match to in vivo data.

The challenges of Raman microspectroscopy lie in the fact that the peak assignment to chemical interactions and further to biochemical structures is challenging and has to be conducted with great care. Moreover, the current instrumentation and data analysis require higher speed and simplification, since only thereby Raman microspectroscopy will become widely applicable to biologists and clinicians not specialized in biophotonics. These insights will not only help to efficiently identify senescent cells in a label-free and non-destructive manner with large potential for in vivo and ex vivo applications including compound screenings, but also to gain insights into intracellular biochemical changes that occur during aging.

## References

[CR1] Ager JW, Nalla RK, Breeden KL, Ritchie RO (2005). Deep-ultraviolet Raman spectroscopy study of the effect of aging on human cortical bone. J Biomed Opt.

[CR2] Ager JW, Nalla RK, Balooch G, Kim G, Pugach M, Habelitz S (2006). On the increasing fragility of human teeth with age: a deep-UV resonance Raman study. J Bone Miner Res.

[CR3] Akkus O, Polyakova-akkus A, Adar F, Schaffler MB (2003). Aging of Microstructural Compartments in Human Compact Bone. J Bone Miner Res.

[CR4] Ashtikar M, Matthäus C, Schmitt M, Krafft C, Fahr A, Popp J (2013) Non-invasive depth profile imaging of the stratum corneum using confocal Raman microscopy: first insights into the method. Eur J Pharm Sci Elsevier BV 50(5):601–608 10.1016/j.ejps.2013.05.030 23764946

[CR5] Baar MP, Brandt RMC, Putavet DA, Klein JDD, Derks KWJ, Bourgeois BRM et al (2017) Targeted apoptosis of senescent cells restores tissue homeostasis in response to chemotoxicity and aging. Cell 169(1):132–147.e16 10.1016/j.cell.2017.02.031 PMC5556182 28340339

[CR6] Bai H, Li H, Han ZZ, Zhang C, Zhao J, Miao C et al (2015) Label-free assessment of replicative senescence in mesenchymal stem cells by Raman microspectroscopy. Biomed Opt Express 6(11):4493 10.1364/BOE.6.004493 PMC4646556 26601012

[CR7] Baker DJ, Wijshake T, Tchkonia T, LeBrasseur NK, Childs BG, van de Sluis B et al (2011) Clearance of p16Ink4a-positive senescent cells delays ageing-associated disorders. Nature Nature Publishing Group 479(7372):232–236 10.1038/nature10600 PMC3468323 22048312

[CR8] Baker DJ, Childs BG, Durik M, Wijers ME, Sieben CJ, Zhong J, A. Saltness R, Jeganathan KB, Verzosa GC, Pezeshki A, Khazaie K, Miller JD, van Deursen JM (2016) Naturally occurring p16 Ink4a -positive cells shorten healthy lifespan. Nature Nature Publishing Group 530(7589):184–189 10.1038/nature16932 PMC4845101 26840489

[CR9] Beattie JR, McGarvey JJ, Stitt AW (2013) Raman spectroscopy for the detection of AGEs/ALEs. In: Galluzzi L, Vitale I, Kepp O, Kroemer G (eds) Methods Mol Biol, vol 965. Humana Press, Totowa, pp 297–312 10.1007/978-1-62703-239-1_20 23296667

[CR10] Bellantuono I (2018) Find drugs that delay many diseases of old age. Nature Nature Publishing Group 554(7692):293–295 10.1038/d41586-018-01668-0 29446384

[CR11] Bernstein PS, Zhao DY, Wintch SW, Ermakov IV, McClane RW, Gellermann W (2002). Resonance Raman measurement of macular carotenoids in normal subjects and in age-related macular degeneration patients. Ophthalmology.

[CR12] Bitto A, Sell C, Crowe E, Lorenzini A, Malaguti M, Hrelia S et al (2010) Stress-induced senescence in human and rodent astrocytes. Exp Cell Res Elsevier Inc 316(17):2961–2968 10.1016/j.yexcr.2010.06.021 20620137

[CR13] Bocklitz TW, Crecelius AC, Matthäus C, Tarcea N, von Eggeling F, Schmitt M et al (2013) Deeper understanding of biological tissue: quantitative correlation of MALDI-TOF and Raman imaging. Anal Chem 85(22):10829–10834 10.1021/ac402175c 24127731

[CR14] Bodnar AG, Ouellette M, Frolkis M, Holt SE, Chiu CP, Morin GB, Harley CB, Shay JW, Lichtsteiner S, Wright WE (1998) Extension of life-span by introduction of telomerase into normal human cells. Science 279(5349):349–352 10.1126/science.279.5349.349 9454332

[CR15] Bogliolo L, Murrone O, Di Emidio G, Piccinini M, Ariu F, Ledda S (2013). Raman spectroscopy-based approach to detect aging-related oxidative damage in the mouse oocyte. J Assist Reprod Genet.

[CR16] Boireau-Adamezyk E, Baillet-Guffroy A, Stamatas GN (2014). Age-dependent changes in stratum corneum barrier function. Skin Res Technol.

[CR17] Brauchle E, Schenke-Layland K (2013). Raman spectroscopy in biomedicine—non-invasive in vitro analysis of cells and extracellular matrix components in tissues. Biotechnol J.

[CR18] Brauchle E, Noor S, Holtorf E, Garbe C, Schenke-Layland K, Busch C (2014). Raman spectroscopy as an analytical tool for melanoma research. Clin Exp Dermatol.

[CR19] Brauchle E, Thude S, Brucker SY, Schenke-Layland K (2014b) Cell death stages in single apoptotic and necrotic cells monitored by Raman microspectroscopy. Sci Rep 4:4698 10.1038/srep04698 PMC3986703 24732136

[CR20] Buckley K, Kerns JG, Vinton J, Gikas PD, Smith C, Parker AW, Matousek P, Goodship AE (2015). Towards the in vivo prediction of fragility fractures with Raman spectroscopy. J Raman Spectrosc.

[CR21] Butler HJ, Ashton L, Bird B, Cinque G, Curtis K, Dorney J, Esmonde-White K, Fullwood NJ, Gardner B, Martin-Hirsch PL, Walsh MJ, McAinsh MR, Stone N, Martin FL (2016). Using Raman spectroscopy to characterize biological materials. Nat Protoc Nature Publishing Group.

[CR22] Campisi J (2005) Senescent cells, tumor suppression, and organismal aging: good citizens, bad neighbors. Cell 120(4):513–522 10.1016/j.cell.2005.02.003 15734683

[CR23] Campisi J, Andersen JK, Kapahi P, Melov S (2011) Cellular senescence: a link between cancer and age-related degenerative disease? Semin Cancer Biol Elsevier Ltd 21(6):354–359 10.1016/j.semcancer.2011.09.001 PMC3230665 21925603

[CR24] Cardoso AL, Fernandes A, Aguilar-Pimentel JA, de Angelis MH, Guedes JR, Brito MA et al (2018) Towards frailty biomarkers: candidates from genes and pathways regulated in aging and age-related diseases. Ageing Res Rev Elsevier Ltd 47:214–277 10.1016/j.arr.2018.07.004 30071357

[CR25] Charwat V, Schütze K, Holnthoner W, Lavrentieva A, Gangnus R, Hofbauer P et al (2015) Potential and limitations of microscopy and Raman spectroscopy for live-cell analysis of 3D cell cultures. J Biotechnol Elsevier Ltd 205:70–81 10.1016/j.jbiotec.2015.02.007 25687101

[CR26] Choe CS, Schleusener J, Lademann J, Darvin ME (2018) Age related depth profiles of human stratum Corneum barrier-related molecular parameters by confocal Raman microscopy in vivo. Mech Ageing Dev Elsevier Ltd 172:6–12 10.1016/j.mad.2017.08.011 28844969

[CR27] Choi JS, Ilin Y, Kraft ML, Harley BAC (2018). Tracing hematopoietic progenitor cell neutrophilic differentiation via Raman spectroscopy. Bioconjug Chem American Chemical Society.

[CR28] Collado M, Serrano M (2006) The power and the promise of oncogene-induced senescence markers. Nat Rev Cancer 6(6):472–476 10.1038/nrc1884 16723993

[CR29] Coppe JP, Desprez PY, Krtolica A, Campisi J (2010) The senescence-associated secretory phenotype: the dark side of tumor suppression. Annu Rev Pathol 5:99–118 10.1146/annurev-pathol-121808-102144 PMC4166495 20078217

[CR30] Czamara K, Majka Z, Fus A, Matjasik K, Pacia MZ, Sternak M, et al. (2018) Raman spectroscopy as a novel tool for fast characterization of the chemical composition of perivascular adipose tissue. Analyst Royal Society of Chemistry 143(24):5999–6005 10.1039/c8an01307a 30334021

[CR31] Davalos AR, Kawahara M, Malhotra GK, Schaum N, Huang J, Ved U, Beausejour CM, Coppe JP, Rodier F, Campisi J (2013). p53-dependent release of Alarmin HMGB1 is a central mediator of senescent phenotypes. J Cell Biol.

[CR32] de Vasconcelos Nasser Caetano L, de Oliveira Mendes T, Bagatin E, Amante Miot H, Marques Soares JL, Simoes e Silva Enokihara MM (2017). In vivo confocal Raman spectroscopy for intrinsic aging and photoaging assessment. J Dermatol Sci Japanese Society for Investigative Dermatology.

[CR33] Debacq-Chainiaux F, Erusalimsky JD, Campisi J, Toussaint O (2009). Protocols to detect senescence-associated beta-galactosidase (SA-betagal) activity, a biomarker of senescent cells in culture and in vivo. Nat Protoc Nature Publishing Group.

[CR34] Demaria M, O’Leary MN, Chang J, Shao L, Liu S, Alimirah F et al (2017) Cellular senescence promotes adverse effects of chemotherapy and cancer relapse. Cancer Discov 7(2):165–176 10.1158/2159-8290.CD-16-0241 PMC5296251 27979832

[CR35] Dinarelli S, Longo G, Krumova S, Todinova S, Danailova A, Taneva SG, Lenzi E, Mussi V, Girasole M (2018). Insights into the morphological pattern of erythrocytes’ aging: coupling quantitative AFM data to microcalorimetry and Raman spectroscopy. J Mol Recognit.

[CR36] Doty KC, Lednev IK (2018). Differentiating donor age groups based on Raman spectroscopy of bloodstains for forensic purposes. ACS Cent Sci American Chemical Society.

[CR37] Duraipandian S, Traynor D, Kearney P, Martin C, O’Leary JJ, Lyng FM (2018) Raman spectroscopic detection of high-grade cervical cytology: using morphologically normal appearing cells. Sci Rep 8(1):15048 10.1038/s41598-018-33417-8 PMC6177468 30301922

[CR38] Eberhardt K, Beleites C, Marthandan S, Matthäus C, Diekmann S, Popp J (2017). Raman and infrared spectroscopy distinguishing replicative senescent from proliferating primary human fibroblast cells by detecting spectral differences mainly due to biomolecular alterations. Anal Chem.

[CR39] Eberhardt K, Matthäus C, Winter D, Wiegand C, Hipler U-C, Diekmann S (2017). Raman and infrared spectroscopy differentiate senescent from proliferating cells in a human dermal fibroblast 3D skin model. Analyst Royal Society of Chemistry.

[CR40] Egawa M, Tagami H (2008). Comparison of the depth profiles of water and water-binding substances in the stratum corneum determined in vivo by Raman spectroscopy between the cheek and volar forearm skin: effects of age, seasonal changes and artificial forced hydration. Br J Dermatol.

[CR41] Erusalimsky JD, Kurz DJ (2005) Cellular senescence in vivo: Its relevance in ageing and cardiovascular disease. Exp Gerontol Elsevier Ltd 40(8-9):634–642 10.1016/j.exger.2005.04.010 15970413

[CR42] Figueira I, Fernandes A, Mladenovic Djordjevic A, Lopez-Contreras A, Henriques CM, Selman C et al (2016) Interventions for age-related diseases: Shifting the paradigm. Mech Ageing Dev Elsevier Ltd 160:69–92 10.1016/j.mad.2016.09.009 27693441

[CR43] Fumagalli M, Rossiello F, Mondello C, D’Adda Di Fagagna F (2014). Stable cellular senescence is associated with persistent DDR activation. PLoS One.

[CR44] Gamsjaeger S, Masic A, Roschger P, Kazanci M, Dunlop JWC, Klaushofer K et al (2010) Cortical bone composition and orientation as a function of animal and tissue age in mice by Raman spectroscopy. Bone Elsevier Ltd 47(2):392–399 10.1016/j.bone.2010.04.608 20450992

[CR45] GBD 2015 DALYs and HALE Collaborators (2016). Global, regional, and national disability-adjusted life-years (DALYs) for 315 diseases and injuries and healthy life expectancy (HALE), 1990–2015: a systematic analysis for the Global Burden of Disease Study 2015. Lancet (London, England).

[CR46] GBD 2015 Mortality and Causes of Death Collaborators (2016). Global, regional, and national life expectancy, all-cause mortality, and cause-specific mortality for 249 causes of death, 1980–2015: a systematic analysis for the Global Burden of Disease Study 2015. Lancet (London, England).

[CR47] Georgakopoulou EA, Tsimaratou K, Evangelou K, Fernandez Marcos PJ, Zoumpourlis V, Trougakos IP et al (2013) Specific lipofuscin staining as a novel biomarker to detect replicative and stress-induced senescence. A method applicable in cryo-preserved and archival tissues. Aging (Albany NY) 5(1):37–50 10.18632/aging.100527 PMC3616230 23449538

[CR48] Glenn JV, Beattie JR, Barrett L, Frizzell N, Thorpe SR, Boulton ME, McGarvey JJ, Stitt AW (2007). Confocal Raman microscopy can quantify advanced glycation end product (AGE) modifications in Bruch’s membrane leading to accurate, nondestructive prediction of ocular aging. FASEB J.

[CR49] Gniadecka M, Nielsen OF, Wessel S, Heidenheim M, Christensen DH, Wulf HC (1998) Water and protein structure in photoaged and chronically aged skin. J Invest Dermatol Elsevier Ltd 111(6):1129–1133 10.1046/j.1523-1747.1998.00430.x 9856828

[CR50] González FJ, Castillo-Martínez C, Martínez-Escanamé M, Ramírez-Elías MG, Gaitan-Gaona FI, Oros-Ovalle C, Moncada B (2012). Noninvasive estimation of chronological and photoinduced skin damage using Raman spectroscopy and principal component analysis. Skin Res Technol.

[CR51] Gorenne I, Kavurma M, Scott S, Bennett M (2006) Vascular smooth muscle cell senescence in atherosclerosis. Cardiovasc Res 72(1):9–17 10.1016/j.cardiores.2006.06.004 16824498

[CR52] Hayflick L, Moorhead PS (1961) The serial cultivation of human diploid cell strains. Exp Cell Res 25:585–621 10.1016/0014-4827(61)90192-6 13905658

[CR53] Hernandez-Segura A, de Jong TV, Melov S, Guryev V, Campisi J, Demaria M (2017). Unmasking transcriptional heterogeneity in senescent cells. Curr Biol.

[CR54] Ilin Y, Choi JS, Harley BAC, Kraft ML (2015) Identifying states along the hematopoietic stem cell differentiation hierarchy with single cell specificity via Raman spectroscopy. Anal Chem 87(22):11317–11324 10.1021/acs.analchem.5b02537 PMC4687963 26496164

[CR55] Jermyn M, Mok K, Mercier J, Desroches J, Pichette J, Saint-Arnaud K (2015). Intraoperative brain cancer detection with Raman spectroscopy in humans. Sci Transl Med.

[CR56] Kassem M, Marie PJ (2011) Senescence-associated intrinsic mechanisms of osteoblast dysfunctions. Aging Cell 10(2):191–197 10.1111/j.1474-9726.2011.00669.x 21210937

[CR57] Lämmermann I, Terlecki-Zaniewicz L, Weinmüllner R, Schosserer M, Dellago H, de Matos Branco AD, et al. (2018) Blocking negative effects of senescence in human skin fibroblasts with a plant extract. NPJ Aging Mech Dis Nature Publishing Group 4:4 10.1038/s41514-018-0023-5 PMC5895844 29675264

[CR58] Lau K, Hobro A, Smith T, Thurston T, Lendl B (2012) Label-free non-destructive in situ biochemical analysis of nematode Steinernema kraussei using FPA-FTIR and Raman spectroscopic imaging. Vib Spectrosc Elsevier Ltd 60:34–42

[CR59] Lee SH, Kim OK, Lee S, Kim JK (2018) Local-dependency of morphological and optical properties between breast cancer cell lines. Spectrochim Acta A Mol Biomol Spectrosc Elsevier Ltd 205:132–138 10.1016/j.saa.2018.07.024 30015018

[CR60] Li M, Xu J, Romero-Gonzalez M, Banwart SA, Huang WE (2012). Single cell Raman spectroscopy for cell sorting and imaging. Curr Opin Biotechnol.

[CR61] Managò S, Mirabelli P, Napolitano M, Zito G, De Luca AC (2018). Raman detection and identification of normal and leukemic hematopoietic cells. J Biophotonics.

[CR62] Mariani MM, MacCoux LJ, Matthäus C, Diem M, Hengstler JG, Deckert V (2010). Micro-Raman detection of nuclear membrane lipid fluctuations in senescent epithelial breast cancer cells. Anal Chem.

[CR63] Matthäus C, Boydston-White S, Miljković M, Romeo M, Diem M (2006) Raman and infrared microspectral imaging of mitotic cells. Appl Spectrosc 60(1):1–8 10.1366/000370206775382758 PMC2732123 16454901

[CR64] Milovanovic P, von Scheidt A, Mletzko K, Sarau G, Püschel K, Djuric M et al (2018) Bone tissue aging affects mineralization of cement lines. Bone Elsevier Ltd 110:187–193 10.1016/j.bone.2018.02.004 29427789

[CR65] Minamino T (2002) Endothelial cell senescence in human atherosclerosis: role of telomere in endothelial dysfunction. Circulation 105(13):1541–1544 10.1161/01.cir.0000013836.85741.17 11927518

[CR66] Movasaghi Z, Rehman S, Rehman IU (2007). Raman spectroscopy of biological tissues. Appl Spectrosc Rev.

[CR67] Narita M, Narita M, Nũnez S, Heard E, Narita MM, Lin AW (2003). Rb-mediated heterochromatin formation and silencing of E2F target genes during cellular senescence. Cell.

[CR68] Nguyen TT, Happillon T, Feru J, Brassart-Passco S, Angiboust JF, Manfait M, Piot O (2013). Raman comparison of skin dermis of different ages: focus on spectral markers of collagen hydration. J Raman Spectrosc.

[CR69] Notingher I, Jell G, Notingher PL, Bisson I, Tsigkou O, Polak JM (2005). Multivariate analysis of Raman spectra for in vitro non-invasive studies of living cells. J Mol Struct.

[CR70] Obana A, Gohto Y, Tanito M, Okazaki S, Gellermann W, Bernstein PS, Ohira A (2014). Effect of age and other factors on macular pigment optical density measured with resonance Raman spectroscopy. Graefes Arch Clin Exp Ophthalmol.

[CR71] Prats Mateu B, Harreither E, Schosserer M, Puxbaum V, Gludovacz E, Borth N et al (2017) Label-free live cell imaging by Confocal Raman Microscopy identifies CHO host and producer cell lines. Biotechnol J 12(1):1–8 10.1002/biot.201600037 PMC5244663 27440252

[CR72] Pudlas M, Brauchle E, Klein TJ, Hutmacher DW, Schenke-Layland K (2013) Non-invasive identification of proteoglycans and chondrocyte differentiation state by Raman microspectroscopy. J Biophotonics 6(2):205–211 10.1002/jbio.201200064 22678997

[CR73] Raman CV (1928) A new radiation. Indian J Phys 2:387–398

[CR74] Rösch P, Harz M, Peschke K-D, Ronneberger O, Burkhardt H, Popp J (2006) Identification of single eukaryotic cells with micro-Raman spectroscopy. Biopolymers 82(4):312–316 10.1002/bip.20449 16421914

[CR75] Sahu A, Deshmukh A, Ghanate AD, Singh SP, Chaturvedi P, Krishna CM (2012). Raman spectroscopy of Oral buccal mucosa: a study on age-related physiological changes and tobacco-related pathological changes. Technol Cancer Res Treat.

[CR76] Santos IP, Caspers PJ, Bakker Schut TC, Van Doorn R, Noordhoek Hegt V, Koljenović S (2016). Raman spectroscopic characterization of melanoma and benign melanocytic lesions suspected of melanoma using high-wavenumber Raman spectroscopy. Anal Chem.

[CR77] Santos IP, Barroso EM, Bakker Schut TC, Caspers PJ, Van Lanschot CGF, Choi DH (2017). Raman spectroscopy for cancer detection and cancer surgery guidance: translation to the clinics. Analyst.

[CR78] Schosserer M, Grillari J, Breitenbach M (2017) The dual role of cellular senescence in developing tumors and their response to cancer therapy. Front Oncol 7:278 10.3389/fonc.2017.00278 PMC5703792 29218300

[CR79] Shipp DW, Rakha EA, Koloydenko AA, Macmillan RD, Ellis IO, Notingher I (2018). Intra-operative spectroscopic assessment of surgical margins during breast conserving surgery. Breast Cancer Res.

[CR80] Surmacki JM, Woodhams BJ, Haslehurst A, Ponder BAJ, Bohndiek SE (2018). Raman micro-spectroscopy for accurate identification of primary human bronchial epithelial cells. Sci Rep Springer US.

[CR81] Swain RJ, Stevens MM (2007) Raman microspectroscopy for non-invasive biochemical analysis of single cells. Biochem Soc Trans 35(Pt 3):544–549 10.1042/BST0350544 17511648

[CR82] Szafraniec E, Wiercigroch E, Czamara K, Majzner K, Staniszewska-Slezak E, Marzec KM (2018). Diversity among endothelial cell lines revealed by Raman and Fourier-transform infrared spectroscopic imaging. Analyst Royal Society of Chemistry.

[CR83] Tchkonia T, Zhu Y, Van Deursen J, Campisi J, Kirkland JL (2013) Cellular senescence and the senescent secretory phenotype: therapeutic opportunities. J Clin Invest 123(3):966–972 10.1172/JCI64098 PMC3582125 23454759

[CR84] Téllez SCA, Pereira L, Dos Santos L, Fávero P, Martin AA (2015). RM1 semi empirical and DFT: B3LYP/3-21G theoretical insights on the confocal Raman experimental observations in qualitative water content of the skin dermis of healthy young, healthy elderly and diabetic elderly women’s. Spectrochim Acta A Mol Biomol Spectrosc.

[CR85] Terentis AC, Fox SA, Friedman SJ, Spencer ES (2013). Confocal Raman microspectroscopy discriminates live human metastatic melanoma and skin fibroblast cells. J Raman Spectrosc.

[CR86] Toledano M, Toledano-Osorio M, Guerado E, Caso E, Aguilera FS, Osorio R (2018) Biochemical assessment of nanostructures in human trabecular bone: Proposal of a Raman microspectroscopy based measurements protocol. Injury Elsevier Ltd 49 Suppl 2:S11–S21 10.1016/j.injury.2018.07.034 30077357

[CR87] Tomimatsu Y, Scherer JR, Yeh Y, Feeney RE (1976). Raman spectra of a solid antifreeze glycoprotein and its liquid and frozen aqueous solutions. J Biol Chem.

[CR88] Toussaint O, Royer V, Salmon M, Remacle J (2002) Stress-induced premature senescence and tissue ageing. Biochem Pharmacol 64(5–6):1007–1009 10.1016/s0006-2952(02)01170-x 12213599

[CR89] Tramini P, Bonnet B, Sabatier R, Maury L (2001). A method of age estimation using Raman microspectrometry imaging of the human dentin. Forensic Sci Int.

[CR90] Trost TM, Lausch EU, Fees SA, Schmitt S, Enklaar T, Reutzel D, Brixel LR, Schmidtke P, Maringer M, Schiffer IB, Heimerdinger CK, Hengstler JG, Fritz G, Bockamp EO, Prawitt D, Zabel BU, Spangenberg C (2005). Premature senescence is a primary fail-safe mechanism of ERBB2-driven tumorigenesis in breast carcinoma cells. Cancer Res.

[CR91] Vasile E, Tomita Y, Brown LF, Kocher O, Dvorak HF (2001) Differential expression of thymosin beta-10 by early passage and senescent vascular endothelium is modulated by VPF/VEGF: evidence for senescent endothelial cells in vivo at sites of atherosclerosis. FASEB J 15(2):458–466 10.1096/fj.00-0051com 11156961

[CR92] Villaret A, Ipinazar C, Satar T, Gravier E, Mias C, Questel E, et al. (2018) Raman characterization of human skin aging. Ski Res Technol Wiley 0(0):1–7. 10.1111/srt.12643 10.1111/srt.12643 30402919

[CR93] Wiemann SU, Satyanarayana A, Tsahuridu M, Tillmann HL, Zender L, Klempnauer J et al (2002) Hepatocyte telomere shortening and senescence are general markers of human liver cirrhosis. FASEB J 16(9):935–942 10.1096/fj.01-0977com 12087054

[CR94] Xu M, Pirtskhalava T, Farr JN, Weigand BM, Palmer AK, Weivoda MM (2018). Senolytics improve physical function and increase lifespan in old age. Nat Med Springer US.

[CR95] Zhu Y, Tchkonia T, Pirtskhalava T, Gower AC, Ding H, Giorgadze N, Palmer AK, Ikeno Y, Hubbard GB, Lenburg M, O'Hara SP, LaRusso NF, Miller JD, Roos CM, Verzosa GC, LeBrasseur NK, Wren JD, Farr JN, Khosla S, Stout MB, McGowan SJ, Fuhrmann-Stroissnigg H, Gurkar AU, Zhao J, Colangelo D, Dorronsoro A, Ling YY, Barghouthy AS, Navarro DC, Sano T, Robbins PD, Niedernhofer LJ, Kirkland JL (2015) The Achilles’ heel of senescent cells: from transcriptome to senolytic drugs. Aging Cell 14(4):644–658 10.1111/acel.12344 PMC4531078 25754370

[CR96] Zhu Y, Doornebal EJ, Pirtskhalava T, Giorgadze N, Wentworth M, Fuhrmann-Stroissnigg H et al (2017) New agents that target senescent cells: the flavone, fisetin, and the BCL-XL inhibitors, A1331852 and A1155463. Aging (Albany NY) 9(3):955–963 10.18632/aging.101202 PMC5391241 28273655

